# Mechanosignaling in Osteoporosis: When Cells Feel the Force

**DOI:** 10.3390/ijms26094007

**Published:** 2025-04-24

**Authors:** Nuo Chen, Marina Danalache, Chen Liang, Dorothea Alexander, Felix Umrath

**Affiliations:** 1 Department of Orthopedic Surgery, University Hospital Tübingen, 72072 Tübingen, Germany; nuo.chen@med.uni-tuebingen.de (N.C.);; 2 Department of Oral and Maxillofacial Surgery, University Hospital Tübingen, 72076 Tübingen, Germany; dorothea.alexander@med.uni-tuebingen.de

**Keywords:** osteoporosis, mechanosignaling, mechanotransduction, mechanosensitive ion channels, secondary osteoporosis

## Abstract

Bone is a highly mechanosensitive tissue, where mechanical signaling plays a central role in maintaining skeletal homeostasis. Mechanotransduction regulates the balance between bone formation and resorption through coordinated interactions among bone cells. Key mechanosensing structures—including the extracellular/pericellular matrix (ECM/PCM), integrins, ion channels, connexins, and primary cilia, translate mechanical cues into biochemical signals that drive bone adaptation. Disruptions in mechanotransduction are increasingly recognized as an important factor in osteoporosis. Under pathological conditions, impaired mechanical signaling reduces bone formation and accelerates bone resorption, leading to skeletal fragility. Defects in mechanotransduction disrupt key pathways involved in bone metabolism, further exacerbating bone loss. Therefore, targeting mechanotransduction presents a promising pharmacological strategy for osteoporosis treatment. Recent advances have focused on developing drugs that enhance bone mechanosensitivity by modulating key mechanotransduction pathways, including integrins, ion channels, connexins, and Wnt signaling. A deeper understanding of mechanosignaling mechanisms may pave the way for novel therapeutic approaches aimed at restoring bone mass, mechanical integrity, and mechanosensitive bone adaptation.

## 1. Introduction

Mechanosignaling is the process by which cells sense and respond to mechanical forces, translating them into biochemical signals that regulate cellular functions [1]. Mechanotransduction, a central component of mechanosignaling, describes the molecular conversion of mechanical stimuli into biochemical and genetic responses [2]. In the 19th century, the German anatomist and surgeon Julius Wolff formulated Wolff’s Law, according to which bones will adapt to the degree of mechanical loading. According to this principle, increased mechanical stress stimulates bone formation and enhances structural integrity, whereas reduced mechanical loading leads to bone resorption and a decrease in bone mass and strength [3]. It is now well established that bone remodeling is regulated through mechanotransduction, wherein bone cells function as mechanosensors, converting mechanical stimuli into biochemical signals that modulate osteogenic and resorptive activity. Among bone cells, osteocytes are recognized as the principal mechanosensors [4]. Embedded within the mineralized extracellular matrix (ECM), they detect mechanical forces and convert them into biochemical signals through a series of mechanosensitive signaling pathways. These signals are subsequently transmitted to neighboring bone cells via autocrine and paracrine mechanisms. Through autocrine and paracrine mechanisms, osteocytes secrete various signaling molecules in response to mechanical loading. These factors subsequently modulate bone remodeling via key signaling pathways such as Wnt/β-catenin [5], transforming growth factor-beta (TGF-β) [6] Yes-associated protein (YAP), and transcriptional coactivator with PDZ-binding motif (TAZ) [7]. These pathways collectively orchestrate bone adaptation by balancing osteoblast-mediated bone formation and osteoclast-driven resorption, ensuring that bone architecture dynamically responds to mechanical demands. Wolff’s Law provides a mechanistic framework for understanding bone adaptation in response to varying mechanical stimuli, including exercise, disuse, fractures, and orthopedic conditions such as osteoporosis. This principle underscores the role of mechanotransduction in regulating bone remodeling, wherein mechanical loading induces osteogenesis and increased bone mass, while unloading or disuse leads to bone resorption and structural weakening.

Osteoporosis is a systemic skeletal disorder characterized by decreased bone mass and microarchitectural deterioration, which increase bone fragility and fracture risk [8]. According to the World Health Organization (WHO), osteoporosis is diagnosed when bone mineral density (BMD) at the femoral neck is 2.5 standard deviations below that of a young, healthy female reference population [9]. A 2021 meta-analysis estimated the global prevalence of osteoporosis in individuals aged ≥50 years to be 21.7% (95% CI: 18.8–25%). The prevalence was 35.3% (95% CI: 27.9–43.4%) among women and 12.5% (95% CI: 9.3–16.7%) among men [10].

Osteoporosis is caused by impaired bone remodeling, a dynamic and tightly regulated process that involves the balanced actions of bone resorption by osteoclasts and bone formation by osteoblasts. Under physiological conditions, these processes are dynamically regulated to maintain structural integrity and appropriate blood calcium levels. However, various factors, including mechanical disuse, hormonal imbalances, and systemic diseases, can disrupt this balance, tipping it toward excessive bone resorption [11]. As a result, cortical bone thickness diminishes, trabecular bone structures become thinner and disrupted, and skeletal mechanical strength declines, increasing fracture risk [12].

Mechanical stress, though primarily influencing bone remodeling after skeletal maturity, plays a pivotal role in maintaining bone health. The following sections explore the molecular mechanisms of mechanosignaling in bone remodeling and the connections between mechanosignaling and osteoporosis.

## 2. Mechanosensing of Bone Cells

**Figure 1:**
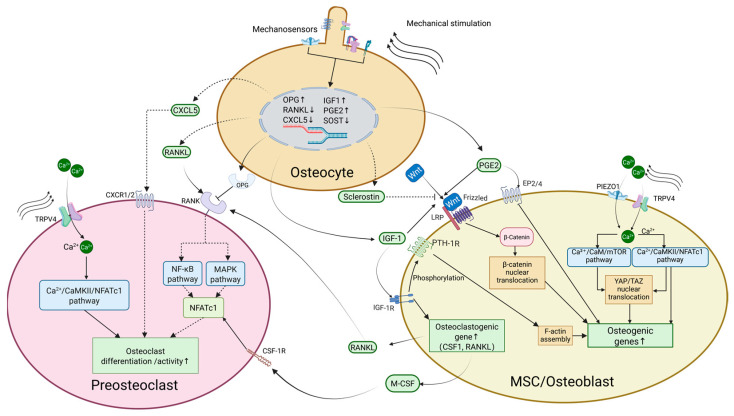
Mechanosensing and signal transduction in bone cells. Osteocytes, as the primary mechanosensitive cells in bone, respond to mechanical stimuli by secretion of signaling molecules such as RANKL, OPG, SOST, IGF-1, PGE2, and CXCL5. Through various signaling pathways, including but not limited to Wnt/β-catenin, YAP/TAZ, and RANKL, they influence the differentiation and function of osteoblasts and osteoclasts. Similarly, osteoblasts and osteoclasts, as well as their progenitors, can also sense mechanical stimuli and regulate the bone remodeling process. Standard arrows indicate stimulatory effects, thin-tailed arrows represent translocation or transport of substances, blocked lines denote inhibitory effects, and dashed arrows signify weakened or reduced effects. Ca^2+^: calcium ion, CaM: calmodulin, CaMKII: calcium/calmodulin-dependent protein kinase II, CSF-1R: colony-stimulating factor 1 receptor, CXCL5: C-X-C motif chemokine ligand 5, CXCR1/2: C-X-C motif chemokine receptor 1/2, EP2/4: prostaglandin E2 receptor 2/4, F-actin: filamentous actin, IGF-1: insulin-like growth factor 1, IGF-1R: insulin-like growth factor 1 receptor, LRP: low-density-lipoprotein receptor-related protein, MAPK: mitogen-activated protein kinase, M-CSF: macrophage colony-stimulating factor, MSC: mesenchymal stem cell, mTOR: mechanistic target of rapamycin, NF-κB: nuclear factor kappa-B, NFATc1: nuclear factor of activated T cells cytoplasmic 1, OPG: osteoprotegerin, PGE2: prostaglandin E2, PIEZO1: Piezo-type mechanosensitive ion channel component 1, PTH-1R: parathyroid hormone 1 receptor, RANK: receptor activator of nuclear factor kappa-Β, RANKL: receptor activator of nuclear factor kappa-Β ligand, SOST: sclerostin, TAZ: transcriptional coactivator with PDZ-binding motif, TRPV4: transient receptor potential vanilloid 4, YAP: Yes-associated protein.

The skeletal response to mechanical stimulation is a highly orchestrated process, with various cells within bone tissue playing specialized roles and influencing each other through multiple pathways. Osteocytes, serving as primary mechanosensors, convert mechanical signals into chemical signals and transmit them to osteoblasts and osteoclasts [13,14]. In addition, osteoblasts, osteoclasts, and their precursor cells also possess intrinsic mechanosensitivity, enabling them to directly detect and respond to mechanical stimuli [15,16,17] (Figure 1).

### 2.1. Osteocytes

Osteocytes, the most abundant cells in mature bone, are embedded within the mineralized matrix and characterized by their dendritic morphology [18]. Their cell bodies reside in lacunae, while their dendritic processes extend through canaliculi [19]. Osteocytes are the primary mechanosensors of bone, detecting interstitial fluid flow within the lacunar–canalicular network caused by mechanical loading [20].

Osteocytes directly perceive mechanical stimulation through various mechanosensors, such as integrins, ion channels, and cilia, and convert these stimuli into biochemical signals that regulate surrounding bone cells. This process involves multiple signaling pathways, among which the Wnt/β-catenin signaling pathway is a key regulator of osteoblast activity and differentiation. Upon binding of Wnt ligands (e.g., Wnt3a) to Frizzled receptors and low-density-lipoprotein receptor-related proteins 5/6 (LRP5/6), the pathway is activated, preventing β-catenin degradation. The accumulated β-catenin then translocates into the nucleus, where it regulates gene expression to promote osteoblast function and bone formation [21]. β-Catenin, a central molecule in the canonical Wnt signaling pathway, directs bone marrow progenitor cells toward osteogenic differentiation by regulating key transcription factors [22]. It also modulates telomerase reverse transcriptase expression to enhance bone-marrow-derived mesenchymal stem cells (BMSC) osteogenesis and inhibit apoptosis [23].

Mechanical unloading increases the secretion of sclerostin, an inhibitor of the Wnt signaling pathway [24]. Sclerostin binds to LRP5/6 on osteoblasts, preventing Wnt ligands from interacting with their receptors, thereby blocking Wnt signaling and suppressing osteoblast differentiation.

Additionally, mechanical stretch stimulation of osteocytes enhances insulin-like growth factor 1 (IGF-1) secretion, thereby promoting mesenchymal stem cell (MSC) proliferation and differentiation [25]. IGF-1 facilitates osteoblast maturation and dendrite elongation by phosphorylating parathyroid hormone 1 receptor (PTH1R) via insulin-like growth factor 1 receptor (IGF1R), which enhances F-actin assembly [26]. Moreover, IGF-1 activates the Wnt3a/LRP5/6 complex, stimulating MSC proliferation and differentiation through the Wnt/β-catenin signaling pathway [27]. On the other hand, IGF-1 can promote the differentiation and maturation of osteoclasts, enhancing bone resorption, by stimulating the expression of M-CSF and RANKL in osteoblasts [28,29].

Mechanical stretch also activates the mechanosensitive ion channel Piezo-type mechanosensitive ion channel component 1 (PIEZO1) in osteocytes, triggering Ca^2+^; influx and promoting TAZ nuclear translocation. This process enhances mitochondrial fission and adenosine triphosphate (ATP) release through ATP dynamin-related protein 1 (DRP1) upregulation [30]. Extracellular ATP acts as a paracrine signal and, following partial degradation to adenosine via CD39 and CD73 ectoenzymes, activates the A2B adenosine receptor (A2BAR) on mesenchymal stem cells (MSCs) [30,31]. This activation elevates intracellular cAMP levels, leading to PKA activation and subsequent phosphorylation of cAMP response element binding protein (CREB). Phosphorylated CREB binds to the *Runx2* promoter, enhancing its transcription [32]. The resulting upregulation of *Runx2*, along with increased *Spp1* expression, promotes osteogenic differentiation.

Similarly, fluid shear stress (FSS)-induced cyclooxygenase-2 (COX-2) expression in osteocytes promotes prostaglandin E2 (PGE2) synthesis, which plays a crucial role in bone adaptation to mechanical load [33,34]. Osteocyte-derived PGE2 is released into the bone matrix through connexin-43 (Cx43) hemichannels in response to FSS [35]. Acting via prostaglandin E receptor (EP) 2 and EP4 receptors, PGE2 enhances osteoblast function by upregulating osteocalcin and downregulating sclerostin, thereby reinforcing the osteogenic response to mechanical loading [35,36]. Mechanistically, PGE2 promotes osteogenesis through receptor-specific MAPK cascades: EP2 activates the cAMP/PKA/p38 MAPK pathway, while EP4 engages ERK and JNK via cAMP and PKC signaling. Both pathways converge on *RUNX2* upregulation, driving osteoblast differentiation and bone formation [37]. Furthermore, in an animal study using mice, PGE2 modulates osteoblast adhesion and alignment by regulating focal adhesion-related genes, including tyrosine kinase (*Src*), Cofilin1 *(Cfl1)*, and Paxillin *(Pxn)*, as well as integrin genes (*Itga5*, *Itgav*, and *Itgb3*) via EP2/EP4 signaling [38].

Apart from direct osteogenic effects, PGE2 also contributes to osteoclast differentiation by increasing the receptor activator of nuclear factor kappa-B ligand (RANKL)/osteoprotegerin (OPG) ratio through activation of the EP2 and EP4 receptors, which signal via the Gαs protein to stimulate the cAMP–PKA pathway in osteoblastic cells [39]. At elevated concentrations, PGE2 enhances RANKL expression in osteocytes while suppressing OPG production, thereby promoting osteoclastogenesis and bone resorption [40,41].

Osteoclast differentiation and function are also regulated by osteocyte-mediated mechanical signaling via the RANK–RANKL–OPG axis [42]. The Notch receptor 3 (NOTCH3) signaling pathway is one of the key mechanisms by which osteocytes respond to mechanical stimulation to regulate the RANKL/OPG ratio [43]. In vivo, NOTCH3, a transmembrane receptor, undergoes ligand-induced cleavage, releasing Notch intracellular domain (NICD). The NICD translocates to the nucleus and forms a complex with recombination signal binding protein for immunoglobulin kappa J region (RBPJ), which regulates the transcription of *Tnfsf11* (RANKL) and *Tnfrsf11b* (OPG) [43]. Mechanical stimulation via FSS inhibits the NOTCH3 signaling pathway through the activation of the PIEZO1 receptor on osteocytes, thereby upregulating OPG expression while suppressing RANKL, ultimately attenuating osteoclastogenesis [44]. FSS also stimulates PTH1R on the primary cilia of MLO-Y4 osteocytes, activating the Hedgehog (GLI), PKA, and protein Kinase C (PKC) signaling pathways. This signaling cascade reduces the secretion of key cytokines, including C-X-C motif chemokine ligand 5 (CXCL5) and interleukin-6 (IL-6), which influence the migration and differentiation of osteoclast precursors, respectively [45,46]. Conversely, mechanical unloading increases RANKL expression and elevates the RANKL/OPG ratio, thereby enhancing osteoclast formation and bone resorption [24,47].

Overall, osteocytes act as central mediators of bone adaptation to mechanical loading, integrating multiple mechanosensitive pathways to regulate both osteoblast and osteoclast activity. Through a complex interplay of signaling molecules such as Wnt, IGF-1, and PGE2, mechanical stimuli orchestrate bone formation while simultaneously modulating bone resorption, ensuring skeletal homeostasis (Figure 1).

### 2.2. Osteoblasts

Osteoblasts, including their progenitors (MSCs), directly respond to mechanical stimuli [48,49]. Unlike Wnt/β-catenin signaling, which is predominantly activated by osteocyte-derived signals, direct mechanical loading triggers distinct intracellular pathways within osteoblasts, primarily through calcium signaling.

FSS, often generated in vivo by small deformations induced by skeletal loading, activates PIEZO1, a mechanosensitive ion channel, leading to rapid calcium influx. This initiates multiple downstream pathways: Ca^2+^ binds to calmodulin (CaM), forming a complex that activates mechanistic target of rapamycin (mTOR), thereby promoting OPG production [50]. Simultaneously, Ca^2+^ activates calcineurin, which dephosphorylates nuclear factor of activated T cells cytoplasmic 1 (NFATc1), facilitating its nuclear translocation and directly upregulating Yes1-associated transcriptional regulator (YAP1) expression. YAP1 forms a complex with β-catenin, enhancing the osteogenic effects of the Wnt signaling pathway. Meanwhile, YAP1 also binds to TEA domain transcription factor (TEAD), promoting the expression of osteogenic genes such as *Sp7* (osterix) and *Spp1* [51] (Figure 1).

Beyond immediate gene regulation, FSS-induced calcium oscillations influence mitochondrial Ca^2+^ uptake, activating the tricarboxylic acid (TCA) cycle and supporting osteoblast metabolism and proliferation [15]. The magnitude, frequency, and duration of shear stress can determine the extent of these responses [52]. Additionally, pulsating fluid flow enhances F-actin formation and upregulates paxillin and integrin expression, improving osteoblast adhesion and responsiveness to external mechanical cues [53].

Hydrostatic pressure (HP), another form of mechanical loading, supports osteogenic factor expression in BMSCs while preserving cytoskeletal integrity under prolonged stress [54]. Compared to FSS, HP better sustains a robust microtubule and F-actin network during extended mechanical stimulation, which may explain why osteoblasts exhibit reduced mechanosensitivity under long-term loading conditions.

### 2.3. Osteoclasts

Osteoclasts and their precursors also exhibit mechanosensitivity, although osteocyte-mediated signaling remains the primary regulator of osteoclast differentiation and activity. It has been shown that FSS stimulates bone-marrow-derived osteoclast progenitor cells to release signaling molecules such as nitric oxide (NO), PGE2, and prostaglandin I2 [16], which regulate osteoblast and osteoclast differentiation and function. Distinct mechanotransduction mechanisms operate during osteoclast maturation. For instance, stromal interaction molecule 1 (STIM1) and transient receptor potential cation channel subfamily V member 4 (TRPV4) mediate calcium influx during the early and late stages of osteoclast differentiation, respectively [17] (Figure 1). Specifically, the opening of TRPV4 induces Ca^2+^ influx, which acts as a second messenger to activate calcineurin, which dephosphorylates the osteoclast-specific transcription factor NFATc1. The dephosphorylated NFATc1 translocates into the nucleus, where it induces the expression of osteoclastogenesis-related genes, including *Acp5* (acid phosphatase 5, tartrate resistant) and *Fos* (Fos proto-oncogene, AP-1 transcription factor subunit) [55], further promoting osteoclast differentiation (Figure 1). As osteoclast differentiation progresses, larger, multinucleated osteoclasts exhibit reduced sensitivity to mechanical stimuli, suggesting that mechanical responsiveness diminishes during differentiation [56].

Recent research shows that FSS influences osteoclast differentiation in hematopoietic progenitor cells. High shear stress (~3 Pa) promotes ATP release and bone resorption, while low shear stress (~0.7 Pa) enhances Piezo1 and ATPase sarcoplasmic/endoplasmic reticulum Ca^2+^ transporting 2 (Atp2a2) expression, providing osteoprotective effects [57]. Moreover, prolonged high shear stress further increases osteoclast formation [57].

Interestingly, FSS gradients influence preosteoclast migration by activating mechanosensitive ion channels, directing cells toward regions of lower stress [58,59]. As the FSS levels inside bone microdamage are lower compared to those on the bone surface [58], this difference may explain the phenomenon of targeted bone remodeling.

## 3. Mechanosensitive Structures Mediating Bone Mechanotransduction

**Figure 2:**
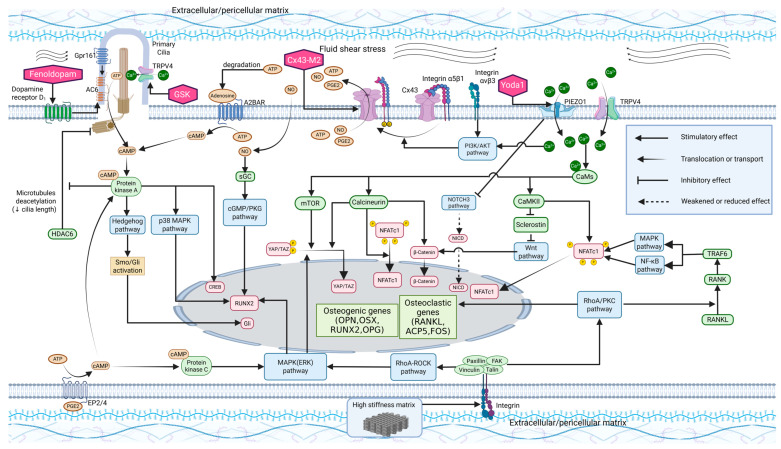
Mechanosensitive structures and their downstream pathways in the bone remodeling process. Mechanical stimuli are transmitted through the extracellular and pericellular matrix, sensed by primary cilia, ion channels, integrins, or connexons, and translated into biological signals through signaling cascades to modulate osteogenic and osteoclastogenic gene expression. Additionally, agents targeting specific mechanoreceptors are shown (pink), which can enhance osteogenic effects. Standard arrows indicate stimulatory effects, thin-tailed arrows represent translocation or transport of substances, blocked lines denote inhibitory effects, and dashed arrows signify weakened or reduced effects. A2BAR: A2B adenosine receptor, AC6: adenylyl cyclase 6, ACP5: acid phosphatase 5, AKT: protein kinase B, ATP: adenosine triphosphate, Ca^2+^: calcium ion, CaM: calmodulin, CaMKII: calcium/calmodulin-dependent protein kinase II, cAMP: cyclic adenosine monophosphate, cGMP: cyclic guanosine monophosphate, CREB: cAMP response element binding protein, Cx43: connexin 43, Cx43-M2: Cx43 hemichannel agonist, EP2/4: prostaglandin E2 receptor 2/4, ERK: extracellular signal-regulated kinase, FAK: focal adhesion kinase, FOS: proto-oncogene c-Fos, Gli: GLI family zinc finger protein, GPR161: G-protein-coupled receptor 161, GSK: GSK1016790A (TRPV4 agonist), HDAC6: histone deacetylase 6, MAPK: mitogen-activated protein kinase, mTOR: mechanistic target of rapamycin, NFATc1: nuclear factor of activated T cells 1, NF-κB: nuclear factor kappa-B, NICD: Notch intracellular domain, NO: nitric oxide, NOTCH3: Notch receptor 3, OPG: osteoprotegerin, OPN: osteopontin, OSX: osterix, PGE2: prostaglandin E2, PI3K: phosphoinositide 3-kinase, PIEZO1: Piezo-type mechanosensitive ion channel component 1, PKA: protein kinase A, PKC: protein kinase C, PKG: protein kinase G, RANK: receptor activator of nuclear factor kappa-B, RANKL: receptor activator of nuclear factor kappa-B ligand, RhoA: Ras homolog family member A, ROCK: Rho-associated protein kinase, RUNX2: Runt-related transcription factor 2, sGC: soluble guanylyl cyclase, Smo: Smoothened, TAZ: transcriptional co-activator with PDZ-binding motif, TRAF6: TNF receptor-associated factor 6, TRPV4: transient receptor potential cation channel subfamily V member 4, YAP: Yes-associated protein, Yoda1: PIEZO1 agonist.

Mechanosensitive structures within bone cells and their surrounding extracellular environments serve as key transducers, converting mechanical forces into biochemical signaling cascades that regulate bone remodeling and adaptation. Essential mechanosensitive components include the ECM as well as pericellular matrix (PCM), integrins, ion channels, connexons, and primary cilia, each contributing to the transduction of mechanical stimuli into cellular responses (Figure 2).

### 3.1. Extracellular Matrix (ECM) and Pericellular Matrix (PCM)

In bone tissue, the ECM is primarily generated through the deposition and subsequent mineralization of the organic matrix secreted by osteoblasts during bone formation [60]. This highly organized network is critical for distributing, absorbing, and transmitting mechanical stresses across bone tissue at both macroscopic and microscopic scales. The ECM consists of organic components—including collagen, non-collagenous proteins, and proteoglycans—and inorganic components, predominantly hydroxyapatite crystals [61]. Among these organic components, type I collagen represents approximately 90% of the total collagen content, forming a structured and interconnected fibrous network with other ECM components [62].

Within the ECM, mature osteocytes reside inside an intricate network known as the lacuno-canalicular system (LCS). Here, the osteocytes are surrounded by a specialized, low-mineralized matrix layer termed the PCM, which is approximately 0.5–1.0 μm thick and lines the inner surfaces of the lacunae and canaliculi [4]. A narrow gap of about 50–80 nm exists between the PCM and the osteocyte membrane, facilitating ion exchange, nutrient diffusion, and interstitial fluid flow within the LCS [63]. Therefore, the ECM predominantly transmits macroscopic mechanical loads throughout bone tissue, whereas the PCM directly modulates the mechanosensitivity of osteocytes at the microscopic level, mediating osteocyte responses to mechanical signals and subsequent cellular signaling cascades.

#### 3.1.1. ECM Stiffness and Osteocyte Mechanosensitivity

As the primary mechanosensing cells in bone, osteocytes also exhibit significant changes in morphology, cytoskeletal organization, and intercellular communication in response to ECM stiffness. Osteocytes interact with the ECM via integrins, and changes in matrix stiffness influence cytoskeletal organization and cell morphology. In general, a stiffer ECM promotes osteocyte spreading and the formation of a robust fibrillar cytoskeleton, whereas a softer ECM leads to cell contraction and reduced cytoskeletal tension [64]. Additionally, ECM stiffness modulates fibronectin–integrin interactions, affecting the levels of focal adhesion proteins such as paxillin, which increases with higher ECM stiffness and subsequently enhances Cx43 expression and localization [64]. Upregulated Cx43 may facilitate hemichannel activity and gap junction formation between osteocytes, promoting the secretion of osteogenic factors and enhancing osteocyte mechanosensitivity [35].

Despite the absence of direct evidence in osteocytes, studies from cartilage suggest that ECM stiffness can modulate the activity of mechanosensitive ion channels, such as PIEZO1 and TRPV4, indicating a potential mechanism by which ECM stiffness influences osteocyte mechanotransduction [65,66].

#### 3.1.2. ECM Stiffness and Regulation of Osteoblast and Osteoclast Function and Differentiation

Matrix stiffness of the ECM is a critical determinant of MSC differentiation. During MSC-ECM interactions, a stiffer ECM promotes the maturation of focal adhesion (FA) complexes, facilitating the aggregation of proteins such as talin, vinculin, and focal adhesion kinase (FAK) [64,67]. These FA complexes serve as key sites for mechanosome formation, where mechanosensitive proteins, including integrin-associated signaling molecules, assemble and transduce mechanical cues into intracellular responses [68]. MSCs sense ECM stiffness via integrin/FA signaling, where a rigid ECM (~4.47 kPa) activates the Ras homolog family member A (RhoA)–Rho-associated coiled-coil containing protein kinase (ROCK) pathway, leading to MAPK (ERK and mitogen-activated protein kinase 8 [MAPK8]) signaling within mechanosomes and the nuclear translocation of YAP/TAZ [69]. The mechanosomes shuttle mechanical information to the nucleus, where YAP/TAZ binds to RUNX2, enhancing osteogenic gene transcription. In contrast, a soft ECM (~0.7 kPa) restricts TAZ to the cytoplasm, downregulating osteogenic genes while promoting adipogenic differentiation [69]. Interestingly, another study found that a softer collagen substrate (286 Pa vs. 957 Pa) combined with low cell density (10^3^ cells/cm^2^ vs. 10^4^ cells/cm^2^) promotes osteoblast differentiation into an osteocyte-like phenotype [70]. This suggests that mechanosome-mediated mechanotransduction may play distinct roles at different stages of osteogenic differentiation—a higher ECM stiffness favors early MSC commitment to osteogenesis, while a slightly lower stiffness may facilitate late-stage differentiation and osteocyte maturation.

Osteoclast differentiation is also regulated by substrate stiffness. Compared to soft substrates (~0.1 MPa or lower), a stiffer substrate (~4.05 MPa) accelerates the fusion of preosteoclasts and promotes the formation of larger multinucleated cells [71]. Transcriptomic and immunological analyses indicate that increased substrate stiffness enhances osteoclast differentiation and fusion by activating fibronectin–integrin αvβ3 binding, which triggers downstream signaling pathways involving paxillin, FAK, PKC, and RhoA. The activation of the FAK/paxillin axis not only promotes cytoskeletal remodeling but also enhances RANKL receptor (RANK) signaling efficiency, further amplifying downstream pathways that upregulate key osteoclastogenic genes including *CTSK* (cathepsin K) [71].

#### 3.1.3. Effects of PCM Components on Osteocyte Mechanotransduction

Current research investigating the roles of specific PCM components in osteocyte mechanotransduction has primarily focused on hyaluronic acid and perlecan/heparan sulfate proteoglycan 2 (HSPG2). Hyaluronic acid, a key PCM component, plays a critical role in maintaining osteocyte mechanosensitivity. In vitro studies have shown that hyaluronidase-mediated degradation of PCM surrounding osteocyte dendrites impairs mechanically induced hemichannel opening, suggesting its role in mechanotransduction [72]. Additionally, hyaluronidase treatment leads to the loss of integrin α5, indicating that integrin anchoring may be associated with hyaluronic acid within the PCM [73]. Notably, age-related reductions in PCM hyaluronic acid content have been observed in osteocytes from aged mice, potentially contributing to the decline in osteocyte mechanosensitivity [74].

Perlecan/HSPG2 is a large proteoglycan abundantly present in the PCM [75]. It serves as a structural tether linking the osteocyte membrane to the LCS walls, playing a crucial role in mechanosensation and mechanotransduction [76]. Studies have shown that perlecan deficiency leads to a sparser PCM fiber network. Hydrodynamic analysis indicates that increased PCM fiber spacing weakens its filtering function for large solutes, resulting in enhanced solute transport within the LCS and a 34% increase in FSS. However, the drag force exerted by tethering elements (PCM fibers) decreases by approximately 35% [77]. In vivo tibial loading experiments further revealed that perlecan-deficient mice exhibit diminished osteogenic responses to mechanical stimulation, suggesting an overall reduction in osteocyte mechanosensitivity [77]. To further investigate the impact of perlecan deficiency on osteocyte mechanotransduction, Pei et al. [78] performed real-time Ca^2+^ imaging in situ under cyclic loading. Compared to wild-type mice, perlecan-deficient osteocytes exhibited reduced calcium signaling, with significant decreases in the Ca^2+^ response rate (−58%), calcium peaks (−33%), cells with multiple peaks (−53%), peak magnitude (−6.8%), and the speed of recovery to baseline (−23%).

### 3.2. Integrins and Connexons

Integrins are heterodimeric transmembrane receptors that anchor cells to the ECM, playing a crucial role in maintaining cellular structure, mechanosignaling, and adhesion [79]. In osteocytes, integrins are critical mechanical receptors, particularly integrins α5β1 and αvβ3, which are essential for osteocyte function [80]. Integrin αvβ3, located predominantly on the dendritic processes of osteocytes, facilitates their attachment to the canalicular wall, making these processes more sensitive to mechanical stimuli than the cell body itself [81]. FSS within the lacunar–canalicular network activates integrin αvβ3, triggering the phosphoinositide 3-kinase (PI3K)–protein kinase B (PKB, also known as AKT) pathway. This activation leads to the conformational activation of integrin α5β1. Concurrently, AKT phosphorylates connexin 43 (Cx43), and the activated integrin α5β1 interacts with Cx43, causing the opening of Cx43 hemichannels and the subsequent release of bone morphogenetic factors such as prostaglandin E2 (PGE2) and nitric oxide (NO) [82,83] (Figure 2).

In osteocyte-specific knockouts of integrin β1 and β3 in mice, using Dmp1-Cre (Cre recombinase driven by the dentin-matrix-protein-1 promoter, active in late osteoblasts/osteocytes), the absence of these integrins led to abnormal osteocyte morphology, decreased bone strength, and impaired osteogenic responses to mechanical loading [84,85]. Additionally, integrin β1 deficiency resulted in the enlargement of the lacunar–canalicular system and the shortening and disorientation of type I collagen fibers in long bones [84].

Connexons are transmembrane hemichannels composed of connexin hexamers that pair with connexons from adjacent cells to form gap junctions, enabling the transfer of small molecules and facilitating electrical and chemical intercellular communication [86]. Unpaired connexons also mediate cytoplasmic communication with the extracellular microenvironment by releasing small molecules into the ECM. Among the connexins, Cx43 is the most extensively studied and is crucial in bone metabolism [87]. The absence of Cx43 expression in mouse models leads to delayed osteoblast differentiation, abnormal cranial development, and increased osteoclast proliferation [88]. On the other hand, in aged mice, Cx43 overexpression reduces osteocyte apoptosis, enhances bone formation, and increases bone strength by promoting osteoblast activity and decreasing the number of osteoclasts [89].

Following mechanical stimulation, osteocytes integrate signals through mechanoreceptors such as integrins α5β1 and αvβ3, which open Cx43 hemichannels, allowing the release of bone-synthesis factors like PGE2, NO, and ATP [35,90,91]. PGE2 plays a complex role in this process as it induces the upregulation of Cx43 expression and enhances gap junction functionality [35]. However, prolonged exposure to FSS causes sustained accumulation of extracellular PGE2, which triggers the extracellular signal-regulated kinase 1/2 (ERK1/2) pathway, leading to the phosphorylation of Cx43 and subsequent closure of the hemichannels [92]. Additionally, FSS promotes hemichannel formation by upregulating 14-3-3θ protein levels in MLO-Y4 cells, and this protein binds to Cx43 and integrin α5β1 in the Golgi apparatus, facilitating their assembly into hemichannels on the cell membrane [93].

### 3.3. Mechanosensitive Ion Channels

Mechanosensitive ion channels enable bone cells to perceive and respond to mechanical forces by facilitating ion flux across cell membrane. These channels convert mechanical stimuli into electrical and biochemical signals [94], influencing bone cell behavior. Among the different types of mechanosensitive ion channels, Piezo channels and transient receptor potential channels, in particular TRPV4, are prominently involved in bone metabolism [95,96,97,98].

#### 3.3.1. Piezo Channels

Piezo-type mechanosensitive ion channel components 1 and 2 (PIEZO1 and PIEZO2) are mechanically activated cation channels that mediate calcium influx in response to mechanical stimuli [51,99]. In the human body, PIEZO1 is primarily expressed in non-neuronal cells, whereas PIEZO2 is predominantly expressed in neurons [100]. Both PIEZO1 and PIEZO2 possess a trimeric structure that resembles a three-bladed propeller [101]. Their transmembrane region forms a nano-bowl configuration, providing high sensitivity to mechanical forces, such as membrane tension and curvature changes [101]. Changes in membrane tension, induced by extracellular stimuli, activate Piezo channels, leading to the influx of extracellular cations (e.g., calcium ions) [51]. This, in turn, results in the subsequent activation of various signaling pathways, including the Yes-associated protein (YAP)/TAZ pathway [102], the PI3K/AKT pathway [103], the Ca^2+^/CaM/mTOR pathway, and the calcineurin/NFATc1/Yap1/β-catenin pathway [51] (Figure 2).

In order to investigate the role of PIEZO1 in osteocyte mechanotransduction, Li et al. compared the gene expression of MLO-Y4 cells under static conditions and FSS stimulation [104]. Their findings revealed that *Piezo1* expression was significantly increased in cells subjected to FSS stimulation. Moreover, the study revealed that the knockout of *Piezo1* mRNA in MLO-Y4 cells led to a substantial attenuation of the intracellular calcium increase induced by FSS [104], suggesting that PIEZO1 is involved in the response of MLO-Y4 cells to FSS. Furthermore, researchers observed that *Piezo1* conditional knockout (CKO) mice, with deletion in osteoblasts and osteocytes, showed reduced BMD, cortical thickness, and trabecular number and thickness compared to controls. The bone formation response to mechanical loading was also significantly attenuated in *Piezo1* CKO mice [104]. Notably, the expression of *Piezo1* in cortical bone decreases with age, and cortical bone thickness in *Piezo1* CKO mice also declines noticeably with age [50]. Furthermore, under mechanical loading, PIEZO1 in osteoblasts plays a role in mediating the regulation of osteoclast differentiation. In a study by Wang et al., *Piezo1* CKO mice demonstrated increased bone resorption under mechanical loading conditions, while exhibiting reduced bone loss and osteoclast activation during unloading [105]. In co-culture experiments, *Piezo1*-deficient osteoblasts led to higher osteoclast numbers and resorptive activity compared to controls. This effect may be attributed to a reduction of YAP-dependent type II and IX collagen expression by osteoblasts under mechanical loading, which promotes osteoclast differentiation [105]. Additionally, the mechanism by which PIEZO1 regulates bone metabolism involves the upregulation of OPG through the Ca^2+^/CaM/mTOR signaling pathway, thereby controlling osteoclast formation [50].

Compared to PIEZO1, the expression of PIEZO2 is lower in the human skeleton [100]. In order to investigate their roles in skeletal development, Zhou et al. [51] studied mice with *Piezo1* and/or *Piezo2* deletion in the early limb bud mesenchyme. The study revealed that *Piezo1* CKO mice exhibited multiple fractures in the radius and ulna, along with shortened long bones. In contrast, the study found that the development of the skeleton was normal in *Piezo2* CKO mice. Notably, *Piezo1/2* double-knockout mice manifested more severe skeletal defects than *Piezo1* CKO mice, including additional femoral fractures and further shortening of long bones [51]. These findings suggest that PIEZO1 plays a predominant role in bone growth and development, while PIEZO2 contributes synergistically to a lesser extent.

Beyond their individual contributions, PIEZO1 and PIEZO2 exhibit a synergistic interplay in mechanotransduction, as mentioned before. In human chondrocytes, RT-qPCR analysis revealed significant expression of both *PIEZO1* and *PIEZO2*, which synergistically mediate Ca^2+^ influx signaling under high-mechanical-strain conditions (>50%) [106]. Knockdown of *PIEZO1* or *PIEZO2* significantly reduced the Ca^2+^ response induced by high mechanical strain, confirming that PIEZO1/2 collectively contribute to the mechanosensitivity of chondrocytes [106]. This finding underscores a cooperative mechanotransduction mechanism, highlighting the functional crosstalk between PIEZO1 and PIEZO2 in regulating cellular responses to mechanical stimuli.

#### 3.3.2. TRPV4 Channels

TRPV4 is a non-selective, polymodal cation channel that senses various stimuli, including mechanical shear force, membrane tension, temperature, and osmotic pressure [107]. It has been demonstrated that TRPV4 plays a crucial role in mediating the effects of mechanical stress on bone development, joint formation, and bone metabolism processes [55,96,108,109,110].

Defects in the TRPV4 gene have been associated with various genetic bone diseases, which are characterized by decreased bone density, long bone distortion, spinal curvature, and irregular endochondral ossification [111].

In MSCs, TRPV4 localizes to areas of high strain, specifically to the primary cilium [110], mediating calcium signaling and the expression of early osteogenic genes, such as *SP7*, prostaglandin-endoperoxide synthase 2 (*PTGS2*), and *SPP1*, in response to oscillatory fluid shear [112] (Figure 2). In osteocytes, TRPV4 responds to mechanical stimulation indirectly via NADPH oxidase (NOX2/NOX4)-mediated reactive oxygen species (ROS) production, leading to Ca^2+^ influx. The resulting Ca^2+^-CaM complex activates calcium/calmodulin-dependent protein kinase II (CaMKII) and sclerostin degradation and promotes bone formation. The initial Ca^2+^ peak establishes a foundation for subsequent oscillations, which regulate *Sost*, *Tnfrsf11b*, and *Sp7* expression [113] (Figure 2). Yoneda et al. [114] simultaneously investigated PIEZO1 and TRPV4 in MC3T3-E1 cells (pre-osteoblastic cell line) and found that Yoda1, a PIEZO1 agonist, not only directly activates PIEZO1, but also induces TRPV4-dependent Ca^2+^ signaling. This suggests that in cells with high expression of both channels, PIEZO1 activation may synergistically interact with the TRPV4 pathway to generate additional signaling effects. TRPV4 also facilitates late-stage osteoclast differentiation by mediating calcium influx, replacing early RANKL-induced calcium oscillations [17]. The resulting Ca^2+^/calcineurin/NFATc1 signaling promotes NFATc1 nuclear translocation and osteoclastogenic gene expression [55].

Current studies suggest that targeting TRPV4 could be a promising therapeutic approach for osteoporotic conditions. Mouse studies have demonstrated that *Trpv4* knockout increases trabecular bone volume, reduces osteoclast activity [115], and provides protection against bone loss under conditions such as ovariectomy-induced osteoporosis and mechanical unloading [55,116]. However, there are currently no studies on the effects of TRPV4 antagonists on bone metabolism in human cells.

### 3.4. Primary Cilia

Primary cilia are sensory organelles, capable of detecting and transducing extracellular signals to regulate cellular functions [117]. Primary cilia play a crucial role in mediating the effects of mechanical stimuli on bone development and metabolism. They are present in MSCs, osteoblasts, osteocytes, and macrophages, but not in osteoclasts [118,119,120,121,122]. The formation, maintenance, and function of cilia depend on a process called intraflagellar transport (IFT) [123]. IFT achieves a dynamic balance of components within cilia by regulating bidirectional transport between the cell body and cilia, thereby controlling ciliary length and function [124].

Primary cilia are typically elongated, protrusion-like structures [117]. Within the osteocyte lacuno-canalicular system, their interaction with flowing fluid induces bending and deformation, effectively amplifying small external forces into significant mechanical displacements [125]. This amplification facilitates the activation of mechanosensitive receptors, such as ion channels and G-protein-coupled receptors [110,126], located on the ciliary membrane or adjacent cell membranes. Functioning as a “chemo-signaling nexus”, primary cilia integrate mechanical inputs and coordinate the activation of various signaling pathways, enhancing osteocytes’ responsiveness to mechanical stimuli [117]. Ding et al. found that the length of primary cilia in osteocytes is positively correlated with the release of osteogenic factors under FSS stimulation [127]. Furthermore, under simulated microgravity conditions, changes in IFT-related gene expression lead to shortened cilia in osteocytes, which may partially explain microgravity-induced bone loss and osteocyte dysfunction [118]. These findings offer new insights into potential treatments for weightless osteoporosis.

The activation of mechanosensitive calcium channels like TRPV4 on primary cilia regulates MSC osteogenic differentiation by activating osteoblastogenesis-related pathways [110]. Additionally, FSS sensed via GPR161, a mechanosensitive G-protein-coupled receptor, stimulates adenylyl cyclase 6 (AC6) to generate cAMP and upregulates Hedgehog signaling by promoting Smoothened (Smo) activation and Gli transcription factor modulation. This process synergistically enhances osteogenic gene expression and promotes differentiation [126] (Figure 2). Similarly, in another study, FSS stimulated osteochondroprogenitor cells from mice to upregulate osteogenic markers such as *Spp1*, *Runx2*, and *Bglap* (bone gamma-carboxyglutamate protein or osteocalcin), while this effect was not observed in cells lacking primary cilia [121].

During the differentiation of monocytes into osteoclasts, primary cilia gradually diminish and eventually disappear [122]. Interestingly, treating macrophages with fenoldopam mesylate, a selective dopamine D1 receptor agonist that promotes cilia growth, significantly reduced the expression of osteoclast marker genes and inhibited osteoclast differentiation. However, directly applying mechanical stimulation to macrophages did not notably affect the expression of genes related to osteoclast differentiation [122].

Certain physical therapies for osteoporosis depend on the functional integrity of primary cilia in osteoblasts. Primary cilia sense low-magnitude high-frequency vibration, activating the COX-2/PGE2/EP4 pathway to promote osteogenesis, while the PGE2-EP4 pathway further aids cilia repair during treatment [120]. Additionally, extremely low-frequency electromagnetic fields enhance collagen-1α1 and Wnt10b expression in osteoblasts, inducing osteogenic effects via the Wnt/β-catenin pathway [128].

## 4. Mechanosignaling in Osteoporosis

**Figure 3:**
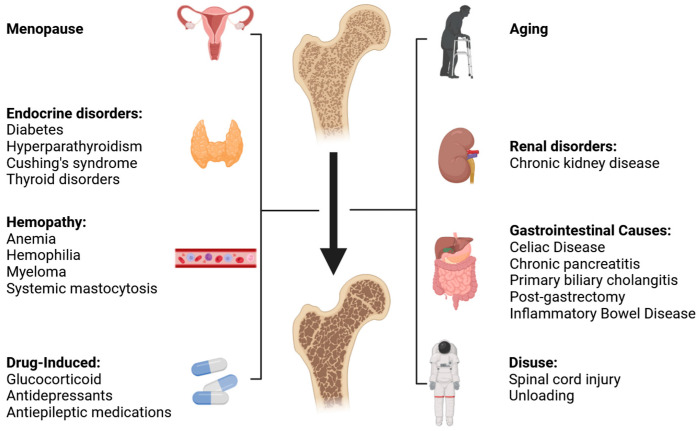
Etiologies of osteoporosis.

Osteoporosis is classified into primary and secondary types based on its etiology and pathogenesis. Primary osteoporosis is characterized by the absence of other identifiable diseases and is further classified into postmenopausal osteoporosis (type 1) and senile osteoporosis (type 2) [129]. Secondary osteoporosis is often attributable to systemic diseases, endocrinopathies, medications, or nutritional deficiencies [130] (Figure 3). Table 1 outlines the major types of osteoporosis and their prevalence rates among individuals with underlying medical conditions.

In disuse osteoporosis, where bone mass is reduced due to insufficient mechanical stimulation, the link between mechanosignaling and bone loss is clear. However, mechanosignaling also plays a role in other etiologies of osteoporosis. Some conditions that cause other forms of osteoporosis can also affect bone cell mechanosignaling. This may contribute to the progression of the disease and can therefore be a potential target for treatment. At the same time, mechanical loading through therapeutic exercise and muscle training is an important factor in the treatment of osteoporosis. Thus, impaired mechanosignaling may also influence the efficacy of classical forms of osteoporosis therapy.

### 4.1. Disuse Osteoporosis

Disuse osteoporosis is a form of secondary osteoporosis that results from skeletal unloading. As such, it underscores the crucial role that mechanical stimuli play in maintaining optimal bone health. Commonly, it can be observed in patients with prolonged bed rest or neuromuscular diseases associated with muscle weakness, such as spinal cord injury (SCI) [157,158] or muscular dystrophy [159]. A compelling example of disuse-related bone loss, albeit without a concurrent osteoporosis diagnosis, can be found in a study of BMD data from cosmonauts aboard the Russian Mir spacecraft. This analysis revealed an average BMD loss of 1–1.5% per month in microgravity [160].

Bone loss in disuse osteoporosis primarily affects weight-bearing bones, but the specific patterns vary depending on the underlying cause. For instance, astronauts exposed to prolonged microgravity experience significant bone loss in weight-bearing bones, including the spine, femoral neck, and lower limbs [161]. By comparison, women with SCI exhibit more pronounced bone loss around the knee joint, while lumbar spine BMD remains relatively preserved [162]. In individuals subjected to long-term bed rest, the greatest BMD loss occurs in the calcaneus (10.4%), followed by the trochanter (4.6%), lumbar spine (3.9%), and femoral neck (3.6%), whereas cranial BMD increases [163]. Unloading, caused by various factors, is the primary contributor to all forms of disuse osteoporosis [164].

#### 4.1.1. Effects of Unloading on Bone Cells

Unloading significantly impairs osteocyte mechanotransduction and cellular viability, primarily through the suppression of key mechanosensitive signaling pathways. In osteocytes, simulated microgravity (SMG) inhibits the Wnt/β-catenin pathway by disrupting F-actin cytoskeletal organization, thereby preventing β-catenin nuclear translocation [24]. Additionally, SMG upregulates sclerostin and zinc finger protein 384 (Znf384/NMP4), both negative regulators of Wnt signaling, further suppressing osteocyte-mediated osteogenic signaling [24].

Moreover, microgravity-induced disruption of the actin cytoskeleton suppresses BMP/Smad signaling in osteocytes [24,165]. Although precise mechanisms remain unclear in osteocytes, related studies in osteoblasts suggest that cytoskeletal depolymerization can block Smad activation via dephosphorylated calponin 1 (CNN1) [165].

Beyond signaling pathway inhibition, SMG also induces endoplasmic reticulum (ER) stress in osteocytes, characterized by morphological alterations in the ER and reduced cellular mechanosensitivity. ER stress activates the unfolded protein response via inositol-requiring enzyme 1 (IRE1)–X-box binding protein 1 spliced form (XBP1s), protein kinase R-like endoplasmic reticulum kinase (PERK)–activating transcription factor 4 (ATF4), and ATF6 pathways, ultimately inducing apoptosis through elevated CHOP (C/EBP homologous protein, a pro-apoptotic transcription factor) expression [166]. Subsequently, apoptotic osteocytes release ATP via pannexin-1 channels, leading to increased RANKL expression in neighboring osteocytes, promoting osteoclast recruitment and subsequent bone resorption [167].

Unloading negatively impacts osteoblast functions primarily through disruptions in extracellular matrix ECM integrity and cytoskeletal organization. Simulated microgravity significantly alters ECM composition, resulting in decreased synthesis of type I collagen and reduced formation of the bone matrix [168,169]. Additionally, changes in proteoglycan and hyaluronan content impair ECM elasticity and mechanosensitive signaling [169,170], while downregulation of critical bone mineralization proteins such as osteonectin and osteocalcin further compromises bone matrix formation and mineralization [171].

ECM integrity is further degraded due to increased expression of matrix metalloproteinases (MMPs) and decreased expression of their inhibitor TIMP3 (tissue inhibitors of metalloproteinases) [169]. Reduced expression of integrin-related genes (e.g., *Itga3*, integrin β1 (*Itgb1*), laminin α3 (*Lama3*)) and adhesion-associated proteins (CD44, CD54, integrin αv/β3) weakens the ECM–cytoskeleton mechanical linkage, significantly decreasing osteoblast adhesion and mechanosensitivity [169]. In parallel, unloading-induced microtubule and F-actin disorganization further compromises osteoblast mechanosensation and function [172,173].

The impact of unloading on osteoclast activity is predominantly mediated indirectly by osteocyte and osteoblast-derived signaling factors, particularly increased receptor activator of nuclear factor-κB ligand (RANKL) expression. Under unloading conditions, osteocyte apoptosis induced by ER stress leads to ATP release and subsequent upregulation of RANKL expression [167]. Additionally, suppression of osteogenic signaling pathways (Wnt/β-catenin and BMP/Smad) elevates the RANKL/OPG ratio, further promoting osteoclast differentiation, activation, and bone resorption [47].

#### 4.1.2. Muscle–Bone Interaction

The musculoskeletal system functions as an integrated unit, where unloading leads to simultaneous muscle and bone loss, with each process exacerbating the other. Beyond the reduction in mechanical stress due to muscle atrophy, muscle-derived bioactive factors actively regulate bone metabolism [174]. Among these factors, irisin, secreted during skeletal muscle contraction, activates the Wnt/β-catenin, MAPK, AMP-activated protein kinase (AMPK), and nuclear factor-κB signaling pathways to promote osteoblast differentiation, inhibit osteoclast activity, and protect osteocytes [175]. Similarly, IGF-1, released during muscle growth and exercise, enhances trabecular and cortical bone formation [176]. Additionally, muscle-derived extracellular vesicles carrying miR-27a facilitate osteogenic differentiation in MC3T3-E1 cells via β-catenin signaling [177]. Moreover, skeletal muscle activity suppresses myostatin secretion, a factor that promotes osteoclast activity by upregulating RANKL expression [174,178]. Given this interplay between muscle and bone, targeting the muscle–bone axis represents a promising approach for preventing and treating disuse osteoporosis.

### 4.2. Postmenopausal Osteoporosis

Postmenopausal osteoporosis, resulting from estrogen deficiency, is the most common type of osteoporosis [12]. In China, approximately 49.3 million women aged 50 and above are affected by osteoporosis, accounting for approximately 29.13% of the population in this age group [25]. A study conducted in India in 2022 found a prevalence of osteoporosis among postmenopausal women of 30.5% [131]. Estrogen modulates bone metabolism via estrogen receptors (ERα, ERβ, and G-protein-coupled estrogen receptor 1 (GPER1)) to regulate key pathways, including RANKL/OPG, Wnt/β-catenin, and TGF-β/BMP [179,180]. In the context of this review, it is noteworthy that estrogen also increases osteocyte mechanosensitivity. Studies have shown that estrogen depletion disrupts osteocyte mechanosomes, specialized multiprotein complexes responsible for mechanotransduction, by reducing their number and organization. These mechanosomes consist of pannexin 1 (Panx1) channels, P2X7 purinergic receptors, and Cav3 voltage-gated Ca^2+^ channels clustered around αvβ3 integrin foci on osteocyte processes [181,182,183].

Furthermore, estrogen deficiency weakens protective mechanisms against load-induced membrane damage, possibly as a result of the loss of estrogen’s antioxidant effects, together diminishing osteocyte responsiveness to mechanical stimuli [184].

### 4.3. Senile Osteoporosis

A meta-analysis in China found an overall osteoporosis prevalence of 18.9% among individuals aged ≥ 60 years, with rates climbing from 15.9% (ages 60–69) to 25.0% (ages 70–79) and 35.6% (ages ≥ 80) [132]. The potential mechanisms of senile osteoporosis involve hormonal changes, such as reductions in sex hormones, vitamin D, and PTH [185]. Furthermore, aging alters the differentiation potential of BMSC, favoring adipocyte formation over osteoblasts, a shift mediated by signaling pathways such as BMP, Wnt, and Notch [186]. Recent studies have demonstrated that aging impairs osteocyte mechanotransduction, contributing to bone deterioration. For example, PIEZO1 expression in human cortical bone declines with age, reducing bone adaptability. This may result from a weakened PIEZO1-mediated upregulation of OPG via the Ca^2+^/CaM/mTOR axis, thereby accelerating bone resorption [50].

Additionally, the accumulation of age-related advanced oxidation protein products induces NOX2-mediated ROS production, leading to Cx43 downregulation and the suppression of osteogenic signaling. The loss of Cx43 has been demonstrated to weaken anti-apoptotic signaling, thereby promoting osteocyte apoptosis and further exacerbating the age-related bone metabolism imbalance [187].

### 4.4. Endocrinological Causes

#### 4.4.1. Diabetes Mellitus

Diabetes elevates fragility fracture risk through deleterious effects on bone metabolism, cellular function, and extracellular matrix composition [188]. Up to 19.1% of type 1 diabetes and 37.8% of type 2 diabetes patients develop osteoporosis [133,134,189]. Notably, fracture risk in diabetes does not always correlate with BMD: type 1 diabetes significantly elevates fracture risk with minimal BMD reduction, whereas type 2 diabetes also elevates fracture risk despite often having normal or even increased BMD [190,191]. This discrepancy may be attributed to low bone turnover, increased cortical porosity, the accumulation of advanced glycation end-products (AGEs), sclerostin upregulation, and an increased risk of falls [192]. Among these factors, AGEs play a critical role by impairing osteocyte viability and mechanosensitivity—possibly via increased focal adhesions—and disrupting bone formation by promoting *Sost* mRNA while inhibiting *Wnt3a* and *LRP6* [193,194,195].

In addition, chronic hyperglycemia was shown to impair osteocyte mechanosensitivity. Animal studies have demonstrated that prolonged high glucose exposure (25 mM, 10 days) alters the expression of key purinergic receptors (P2Y2R, P2Y4R, P2X7R) and Panx1 channels, attenuating Ca^2+^ signaling in response to mechanical loading [196]. Additionally, hyperglycemia disrupts load-induced ATP signaling in bone cells, further compromising osteoblast function [197].

#### 4.4.2. Primary Hyperparathyroidism

Primary hyperparathyroidism features hypercalcemia and elevated PTH, driving bone resorption and raising fracture risk [198]. According to a nested case–control study from 2018, approximately 29% of PHPT patients develop osteoporosis [135], characterized by cortical bone loss at sites like the distal forearm and femoral shaft [135]. Under physiological conditions, PTH, in synergy with mechanical stimulation, downregulates sclerostin levels in osteocytes via Wnt signaling, indirectly promoting osteoblast proliferation and differentiation [199,200,201]. However, sustained high PTH levels increase RANKL while suppressing OPG, thereby enhancing osteoclast differentiation and accelerating bone loss [202,203]. Furthermore, continuous infusion of parathyroid-hormone-related protein (1–36) for 11 days in mice induces *Acp5* expression in osteocytes and enlarges the perilacunar/canalicular space, which may reduce osteocyte mechanosensitivity and impair mechanotransduction [204].

### 4.5. Glucocorticoid-Related Osteoporosis

Glucocorticoid (GC)-induced osteoporosis is the most prevalent form of secondary osteoporosis, with a reported prevalence of 47.7% [155]. GC excess—whether exogenous (e.g., long-term GC therapy) or endogenous (e.g., Cushing’s syndrome)—disrupts bone remodeling by suppressing osteoblastogenesis via Wnt inhibitors, enhancing osteoclast activity through RANKL and macrophage colony-stimulating factor (M-CSF) upregulation, and promoting osteocyte apoptosis [205,206,207]. Consequently, up to 17.7% of individuals with Cushing’s syndrome develop osteoporosis [136].

GCs also impair osteocyte mechanotransduction, reducing their ability to sense and respond to mechanical stimuli. GC-induced osteocytic osteolysis leads to the enlargement of osteocyte lacunae and a reduction in the elastic modulus near lacunar surfaces, which may be associated with the upregulation of MMP-2 and MMP-13 [208]. Additionally, dexamethasone alters the ECM composition by suppressing the synthesis and deposition of type I collagen, the primary component of the bone ECM. It upregulates *COL7A1* (collagen type VII alpha 1 chain), *COL8A1* (collagen type VIII alpha 1 chain), and *TIMP4* (tissue inhibitors of metalloproteinases 4), which may influence ECM attachment and degradation [209]. Moreover, GCs affect O-glycosylated components of the ECM, particularly proteoglycans and glycosaminoglycans, reducing ECM structural stability [210]. These changes ultimately compromise bone matrix integrity and may contribute to impaired bone quality, structural stability, and altered mechanotransduction [211].

Moreover, according to the findings from animal studies, glucocorticoids (dexamethasone) have been demonstrated to suppress the expression of *Hes1*, which is a key transcription factor that regulates *Piezo1*. This in turn results in the downregulation of *Piezo1* expression and a subsequent reduction in mechanosensitivity within osteocytes [206]. Notably, the regulation of PIEZO1 may vary significantly across different cell types. A recent study found that in macrophages, GCs upregulate *Piezo1* via the activation of serum/glucocorticoid-regulated kinase 1 (SGK1), increasing intracellular Ca^2+^ influx, which induces cytoskeletal remodeling, ROS production, and ultimately apoptosis [212]. This regulatory pattern contrasts sharply with that observed in osteocytes, highlighting the cell-type-specific effects of GCs on PIEZO1.

## 5. Potential Treatment Strategies Targeting Mechanosignaling

In recent years, the role of mechanosensation and signal transduction in bone remodeling has been the subject of increased research, leading to the development of novel treatment strategies. Based on their mechanisms of action in mechanotransduction, these therapeutics can be categorized into two major classes: agents targeting mechanosensory structures (e.g., cilia, ion channels, Cx43) and modulators of mechanotransduction pathways (e.g., RANKL/OPG axis, SOST/Wnt signaling).

### 5.1. Primary Cilia Modulators

Primary cilia function as mechanosensors for FSS, with their integrity and length closely linked to mechanosensitivity [213]. Fenoldopam, a selective dopamine D1 receptor agonist, is the most extensively studied and most promising agent in this category. It has been shown to restore ciliary morphology and promote primary cilia elongation by enhancing adenylyl cyclase activity [214,215]. Specifically, fenoldopam activates the AC6/cAMP/PKA axis, which inhibits HDAC6 (histone deacetylase 6) activity, leading to increased microtubule acetylation, enhanced cytoskeletal stability, and subsequent primary cilia elongation [214]. In vitro and in vivo studies indicate that fenoldopam enhances osteocyte mechanosensitivity and increases NO release [214], which subsequently activates the cGMP/PKG signaling pathway in osteoblasts, leading to the phosphorylation and nuclear translocation of RUNX2, thereby upregulating MMP-13 transcription and promoting bone differentiation and remodeling [216]. However, fenoldopam alone does not induce significant osteogenic effects, and it requires co-administration with mechanical stimuli such as exercise [214]. Additionally, fenoldopam has been shown to increase the number and length of primary cilia in macrophage-derived preosteoclasts, leading to a significant reduction in osteoclast differentiation and marker expression, thereby counteracting bone resorption [122].

Lithium ions (Li^+^) also promote primary cilia elongation by stabilizing microtubules through GSK3β inhibition, thereby extending the axoneme, the central microtubule-based structure essential for ciliary function and mechanosensing. Additionally, Li^+^ activates Wnt/β-catenin signaling and upregulates cilia-associated proteins (e.g., kinesin family member 3A [Kif3a], intraflagellar transport 88 (Ift88)), further facilitating ciliary growth and enhancing osteocyte mechanosensitivity [127]. However, another study reported that Li^+^ suppresses osteogenic gene expression (*Spp1*, *Runx2*, *Dlx5* (distal-less homeobox 5)) in MSCs, and prolonged exposure may compromise MSC viability, thus questioning its suitability as a therapeutic option for osteoporosis [217].

### 5.2. Ion Channel Modulators

PIEZO1 receptors and TRPV4 channels on osteocytes serve as mechanosensitive ion channels, facilitating Ca^2+^ influx in response to mechanical stimulation, thereby promoting osteogenic signaling. Yoda1, a selective PIEZO1 agonist, is among the most promising pharmacological candidates in this category. In osteocytes, Yoda1 enhances YAP nuclear translocation via PIEZO1, promoting osteogenesis while inhibiting osteoclast activity [7]. Animal studies further demonstrate that Yoda1 activates the Wnt/β-catenin pathway via Piezo1, thereby inducing MSC osteogenic differentiation and mitigating hindlimb-unloading-induced bone loss [218].

GSK1016790A (GSK), a specific TRPV4 agonist, is commonly used in cellular studies to induce Ca^2+^ influx and activate osteogenic signaling pathways. It promotes osteoblast activity and influences cell proliferation via Wnt/β-catenin signaling [114].

### 5.3. Cx43 Hemichannels

Cx43 hemichannels play a crucial role in mechanotransduction by facilitating the release of osteogenic factors, thereby enhancing bone formation and mechanosensitivity [35]. Cx43-M2, a monoclonal antibody agonist of Cx43 hemichannels, promotes hemichannel opening, restoring mechanosignaling in bone under disuse conditions and increasing bone mass following mechanical loading in murine models [219,220]. Mechanistically, Cx43-M2 enhances PGE2 expression and release in osteocytes, thereby stimulating osteogenesis [220]. PGE2 also suppresses SOST expression via the EP4, which relieves Wnt pathway inhibition and further promotes bone formation [220]. Moreover, Cx43-M2 mitigates the hindlimb-suspension-induced increase in Rankl, thereby reducing osteoclast differentiation [220].

Drugs targeting mechanosensory structures represent a promising therapeutic approach due to their dual benefits in promoting bone formation and regulating resorption. By enhancing skeletal responsiveness to mechanical stimuli, these agents can be effectively combined with physical therapies such as vibration therapy or resistance training to achieve synergistic effects [120]. Although no mechanosensitizer-based drugs have received regulatory approval yet, ongoing research underscores their potential as a novel strategy for osteoporosis treatment.

## 6. Conclusions

This review highlights the intricate role of mechanosignaling in bone metabolism and its implications for osteoporosis. The interplay between mechanical forces and cellular responses orchestrates bone remodeling, balancing resorption and formation to maintain skeletal integrity. The disruption of this balance, resulting from unloading or impaired mechanotransduction, plays a substantial role in the pathogenesis of osteoporosis. Advances in understanding mechanosensitive structures such as integrins, ion channels, connexons, and primary cilia have provided critical insights into the mechanisms by which bone cells sense, interpret, and transduce mechanical signals, shaping skeletal adaptation and remodeling. These mechanosensitive pathways interact with key signaling molecules and networks, including Wnt/β-catenin, RANK/RANKL/OPG, and BMP, to regulate bone cell activity. The identification of mechanosensors like PIEZO1 and TRPV4 as pivotal mediators in osteogenesis and osteoclastogenesis opens new avenues for therapeutic intervention. Therapies targeting mechanosensitive pathways, such as modulators of TRPV4 activity or agents that enhance osteocyte mechanosensitivity, may offer novel strategies to prevent or mitigate osteoporosis. Similarly, interventions that mimic mechanical loading, such as vibration therapy or exercise regimens, could serve as adjuncts to pharmacological treatments, promoting bone health through enhanced mechanotransduction.

Future research should elucidate the molecular underpinnings of mechanotransduction in bone, with a particular focus on the interplay between mechanical cues and systemic regulators such as hormones and cytokines. Additionally, there is a need to investigate the role of the extracellular matrix (ECM) and the pericellular matrix (PCM) in transducing mechanical signals, as well as the consequences of alterations in composition and stiffness. A multidisciplinary approach integrating biomechanics, cell biology, and clinical insights holds the potential to drive innovative therapeutic strategies for osteoporosis, ultimately enhancing skeletal health throughout life.

## Figures and Tables

**Table 1:** Osteoporosis types and their corresponding prevalence.

Types of Osteoporosis	Prevalence Rates
Primary Osteoporosis	
Type I (postmenopausal osteoporosis)	29.13–30.50% [25,131]
Type II (senile osteoporosis)	18.9% [132]
Secondary Osteoporosis	
Endocrine disorders	
Diabetes Hyperparathyroidism Cushing’s syndrome Hyperthyroidism Hypothyroidism	27.67–37.8% [133,134] 29% [135] 17.69% [136] 29.3–37.5% [137,138] 9.4% [139]
Renal disorders	
Chronic kidney disease (stages 3–5)	21.3–31.8% [140,141,142]
Gastrointestinal Causes	
Celiac disease Chronic pancreatitis Primary biliary cholangitis Post-gastrectomy Inflammatory bowel disease	14.4% [143] 23.4% [144] 45.5% [145] 38.2% [146] 14.2% [147]
Hemato-Oncological Causes	
Anemia Hemophilia Myeloma Systemic mastocytosis	2.27% [148] 58.7% [149] 24.5–83% [150,151] 18–40% [152,153,154]
Drug-Induced	
Glucocorticoid	47.7% [155]
Antidepressant	23.5% [156]
Disuse	
Spinal cord injury	21.5–43.8% [157,158]

## Abbreviations

The following abbreviations are used in this manuscript:

A2BAR	A2B adenosine receptor
AC6	Adenylyl cyclase 6
ACP5	Acid phosphatase 5, tartrate resistant
AGEs	Advanced glycation end-products
AKT	Protein kinase b
AMPK	AMP-activated protein kinase
ATP	Adenosine triphosphate
ATP2A2	ATPase sarcoplasmic/endoplasmic reticulum Ca^2+^ transporting 2
BGLAP	Bone gamma-carboxyglutamate protein
BMD	Bone mineral density
BMP	bone morphogenetic protein
BMSC	Bone-marrow-derived mesenchymal stem cell
CaM	Calmodulin
CaMKII	Calcium/calmodulin-dependent protein kinase ii
cAMP	Cyclic adenosine monophosphate
CFL1	Cofilin 1
CHOP	C/EBP homologous protein
CKO	Conditional knockout
CNN1	Calponin 1
COL7A1	Collagen type VII alpha 1 chain
COL8A1	Collagen type VIII alpha 1 chain
COX-2	Cyclooxygenase-2
CREB	cAMP response element binding protein
Cx43	Connexin-43
CXCL5	C-X-C motif chemokine ligand 5
DLX5	Distal-less homeobox 5
DMP1	Dentin matrix acidic phosphoprotein 1
DRP1	Dynamin-related protein 1
ECM	Extracellular matrix
EP	Prostaglandin e2 receptor
ER	Estrogen receptors
ERK	Extracellular signal-regulated kinase
F-actin	Filamentous actin
FAK	Focal adhesion kinase
FOS	Fos proto-oncogene, AP-1 transcription factor subunit
FSS	Fluid shear stress
GC	Glucocorticoid
Gli	GLI family zinc finger protein
GPER1	G-protein-coupled estrogen receptor 1
HP	Hydrostatic pressure
IFT	Intraflagellar transport
IFT88	Intraflagellar transport 88
IGF-1	Insulin-like growth factor 1
IGF1R	Insulin-like growth factor 1 receptor
IL	Interleukin
IRE1	Inositol-requiring enzyme 1
ITGA3	Integrin subunit alpha 3
ITGA5	Integrin subunit alpha 5
ITGAV	Integrin subunit alpha V
ITGB1	Integrin subunit beta 1
ITGB3	Integrin subunit beta 3
JAK	Janus kinase
KIF3A	Kinesin family member 3A
LAMA3	Laminin subunit alpha 3
LCS	Lacuna-canaliculi system
LRP5/6	Lipoprotein receptor-related proteins 5/6
MAPK	Mitogen-activated protein kinase
M-CSF	Macrophage colony-stimulating factor
MMP	Matrix metalloproteinase
MSC	Mesenchymal stem cells
mTOR	Mechanistic target of rapamycin
NFATc1	Nuclear factor of activated T cells cytoplasmic 1
NICD	Notch intracellular domain
NMD4	Nuclear matrix protein 4
NO	Nitric oxide
NOTCH3	Notch receptor 3
NOX	NADPH oxidase
OPG	Osteoprotegerin
Panx1	Pannexin-1
PCM	Pericellular matrix
PGE2	Prostaglandin E2
PHPT	Primary hyperparathyroidism
PI3K	Phosphatidylinositol-3-kinase
PIEZO1	Piezo-type mechanosensitive ion channel component 1
PKA	Protein kinase A
PKB	Protein kinase B
PKC	Protein kinase C
PTH	Parathyroid hormone
PTH1R	Parathyroid hormone 1 receptor
PXN	Paxillin
RANK	Receptor activator of nuclear factor kappa-B
RANKL	Receptor activator of nuclear factor kappa-B ligand
RHOA	Ras homolog family member A
ROCK	Rho-associated coiled-coil containing protein kinase
ROS	Reactive oxygen species
RUNX2	Runx family transcription factor 2
SCI	Spinal cord injury
SGK1	Serum/glucocorticoid-regulated kinase 1
SMAD	Smad family member
SMG	Simulated microgravity
SOST	Sclerostin
SP7	Sp7 transcription factor
SPP1	Secreted phosphoprotein 1
SRC	Proto-oncogene tyrosine-protein kinase Src
STIM1	Stromal interaction molecule 1
TAZ	Transcriptional co-activator with PDZ-binding motif
TCA	Tricarboxylic acid
TEAD	TEA domain transcription factor
TGF-β	Transforming growth factor beta
TIMP3/4	TIMP metallopeptidase inhibitor 3/4
TRPV4	Transient receptor potential cation channel subfamily V member 4
WHO	World Health Organization
YAP	Yes-associated protein
YAP1	Yes1-associated transcriptional regulator
ZNF384	Zinc finger protein 384
